# Influence of Zn^+2^ Doping on Ni-Based Nanoferrites; (Ni_1−x_ Zn_x_Fe_2_O_4_)

**DOI:** 10.3390/nano9071024

**Published:** 2019-07-17

**Authors:** Sadaf Bashir Khan, Syed Irfan, Shern-Long Lee

**Affiliations:** 1 Institute for Advanced Study, Shenzhen University, Shenzhen 518060, China; sadafbashirkhan@szu.edu.cn; 2 Key Laboratory of Optoelectronic Devices and Systems of Ministry of Education and Guangdong Province, College of Optoelectronic Engineering, Shenzhen University, Shenzhen 518060, China; syedirfan@szu.edu.cn; 3 Shenzhen Key Laboratory of Advanced Thin Films and Applications, College of Physics and Optoelectronics Engineering, Shenzhen University, Shenzhen 518060, China

**Keywords:** nickel zinc nanoferrites, frequency, lattice constant, zinc substitution, co-precipitation

## Abstract

Nickel zinc nanoferrites (Ni_1−x_Zn_x_Fe_2_O_4_) were synthesized via a chemical co-precipitation method having stoichiometric proportion (x) altering from 0.00 to 1.00 in steps of 0.25. The synthesized nanoparticles were sintered at 800 °C for 12 h. X-ray diffraction patterns illustrate that the nanocrystalline cubic spinel ferrites have been obtained after sintering. The Scherrer formula is used to evaluate the particle size using the extremely intense peak (311). The experimental results demonstrate that the precipitated particles’ size was in the range of 20–70 nm. Scanning electron microscopy (SEM) is used to investigate the elemental configuration and morphological characterizations of all the prepared samples. FTIR spectroscopy data for respective sites were examined in the range of 300–1000 cm^−1^. The higher frequency band ν_1_ was assigned due to tetrahedral complexes, while the lower frequency band ν_2_ was allocated due to octahedral complexes. Our experimental results demonstrate that the lattice constant a_o_ increases while lattice strain decreases with increasing zinc substitution in nickel zinc nanoferrites.

## 1. Introduction

Nanoparticles retain diverse physical physiognomies and chemical characteristics that are different from the corresponding bulk stable state properties. It is due to the quantum size effect, dimensions, surface influence, or quantum tunneling impact [1,2,3]. Previously, metallic nanoparticles have been comprehensively examined due to their theoretical significance and technical importance for wide-range applications in ferrofluids, microwave devices, magnetic materials, lubricants or catalysts, etc. [4,5]. The nanomaterials comprise nanocrystallites and interfaces. The nanocrystalline interface is a gas-like material, which shows neither long- nor short-range order [2,6]. The nanocrystalline material physical and chemical properties have been influenced by the interfacial structure and the interface volume [7,8].

Ferrites history and their applications have been known for several centuries in the past. Generally, ferrites comprise iron oxide as a main constituent, and metal oxides. Ferrites are divided into different categories depending upon the crystal structure, i.e., spinel ferrite, garnet, ortho-ferrite, and hexagonal ferrites, each having its importance [9,10,11]. However, substituted M-type hexaferrite possess promising potential application in advanced technology. These ferrites comprise a hexagonal structure having ferromagnetic nature, i.e., the large total magnetic moment at operating and ambient temperature. More than 90% of permanent magnets are produced all over the world based on this compound. This compound is a deep semiconductor (~109 Ohm*cm) at room temperature with a ferrimagnetic structure and a total magnetic moment of 20 μB in the ground state [12,13,14]. Furthermore, a large spontaneous polarization and multiferroic properties at room temperature recently discovered in barium hexaferrite were substituted by diamagnetic cations [15]. Herewith, the magnetoelectric characteristics of M-type hexaferrite fabricated by a modified ceramic technique are more advanced than those for the well-known room temperature BiFeO_3_ orthoferrite multiferroic [15,16].

Besides this, spinel ferrite nanoparticles have attained a lot of interest due to their unique magnetic, thermal electrical, dielectric, or catalytic properties for high-tech applications in industries as an inductive or capacitive material, ferrofluids, disk recording, microwave absorbers, transformers, electric generators, or electrical device, etc. [17,18,19]. The spinel structure allows the amalgamation of various metallic ions without altering the spinel crystal structure modifying electrical structural, dielectric, or magnetic properties of spinel ferrites via substituting M^2^ ions [20,21,22,23]. The spinel ferrites structure is composed of [M^+2^]_tet_ [Fe^+3^]_octa_O_4_. However, the induction of a third metal ion helps in modifying the distribution of the ions in the spinel assembly. The concentration of third metal ion alters the distribution of Fe^+3^ and M^+2^ ions affecting the magnetic, catalytic electrical, or dielectric properties [24,25,26]. Different ferrite systems such as Mn–Zn, Ni–Zn, or Mg–Mn are very significant for high-tech applications [27,28,29]. Ni–Zn bulk ferrite nanomaterials are the only core materials applicable to high-frequency applications [27,30,31]. The main drawback is that its performance is constrained to 100 MHz due to eddy current at high frequencies. However, this issue is resolved by enhancing material electrical resistivity keeping the saturation magnetization higher. With the elimination of the inter-granular domain wall and processing material in ultrafine particles, one can achieve higher electrical resistivity [32,33]. The ultrafine grain generates grain boundaries that act as a barrier for electron flow, causing a reduction in the eddy current losses [34,35,36,37].

Here in, we demonstrate the synthesis of Zn-substituted Ni-ferrite nanoparticles via chemical co-precipitation method. We discuss the influence of Zn ions on Ni nano ferrites comprehensively. We investigate the effect of Zn^+2^ doping on the structural, AC conductivity, dielectric impedance, and BET surface area with the variation in zinc concentration in Ni_1−x_Zn_x_Fe_2_O_4_ (x = 0, 0.25, 0.5, 0.75, 1) ferrites as a function of frequency and composition at room temperature.

## 2. Materials and Methods

Different methods have been employed to synthesize nanoferrites, such as sol–gel route [12,38,39], co-precipitation [40], hydrothermal technique, ball-mill [41], or micro-emulsion method [42]. Each mode has its impact on the particle size, morphology, catalytic activity, dielectric, or magnetic properties [43]. Herein, nanocrystalline Ni_1−x_Zn_x_Fe_2_O_4_ particles were produced via a chemical co-precipitation technique. Chemical co-precipitation method plays an influential role in governing particle size, chemical homogeneity, and degree of agglomeration. Different parameters influence the magnetization of substituted ferrite nanoparticles synthesized, such as reaction temperature, initial molar concentration or pH of the suspension, etc. [44,45,46]. Ni_1−x_Zn_x_Fe_2_O_4_ ferrite with x varying from 0 to 1 in step of 0.25 were prepared by a co-precipitating aqueous solution of Ni(NO_3_)_2_.6H_2_O, Zn(NO_3_)_2_.6H_2_O and Fe(NO_3_)_2_.9H_2_O in their respective stoichiometry and kept at 85 °C. The solution was then added to NaOH within 15 s under continuous stirring. During the process, pH of the solution was retained at 12. The acquired solution was then heated at 85 °C for an hour until the solution cooled down to room temperature. The attained precipitated particles were washed numerous times with distilled water to eliminate the salt residues and other impurities till pH 7 is achieved. After this, it was dried in an electric oven at 120 °C for 12 h to eradicate the water contents. Finally, the powder samples were sintered in a muffle furnace at 800 °C for 9 h.

The ferrite nanoparticles formation takes place in two steps: first, a co-precipitation step in which the transformation of metal salts into hydroxides occurs. Second is the fertilization step in which conversion of hydroxides into nanoferrites take place. Thus, the fine particles of metal hydroxides were acquired via co-precipitation of metal cations in alkaline medium. The solid solution of metal hydroxides was then transformed to nickel zinc nanoferrite when it was heated in an alkaline medium at 85 °C, which would require a sufficient time to transform metal hydroxides into ferrites. The inclusive chemical reactions involved in the formation of nanoferrites can be written as:

(1 − x)Ni^2+^ + xZn^2+^ + 2Fe^3+^ + 8OH → (1 − x)Ni(OH)_2_ xZn(OH)_2_·2Fe(OH)_3_ (1)

(2) $$(1-x)\mathrm{Ni}(\mathrm{OH}{)}_{2}\;\mathrm{xZn}(\mathrm{OH}{)}_{2}\cdot 2\mathrm{Fe}(\mathrm{OH}{)}_{3}\;\overset{\mathrm{heat}}{\to }\;\mathrm{heat}\;{\mathrm{Ni}}_{1-x}{\mathrm{Zn}}_{x}{\mathrm{Fe}}_{2}O_{4}\;+\;{\mathrm{nH}}_{2}O$$

The characterization of Ni_1−x_Zn_x_Fe_2_O_4_ samples were performed using different analytical methods. The X-ray diffraction (XRD) patterns of the samples were recorded using Cu Kα (λ = 1.54Ả) in 2θ range 15° to 94° at a step size of 0.02°/s using X-ray diffraction (XRD) instrument Rigaku (Austin TX, 77381-5209, USA). The elemental analysis and morphological characterizations of Ni_1−x_Zn_x_Fe_2_O_4_ were performed via high-resolution JSM 6490LA scanning electron microscopy (Diamond Hill Road, Woonsocket, RI 0289, 5 Product Code: 300574, USA). The Fourier transform infrared (FTIR) spectra of samples of Ni_1−x_Zn_x_ Fe_2_O_4_ were recorded with a Perkin Elmer FTIR spectrometer (4-555 Wentworth Street East Oshawa, ON, L1H 3V8, Canada) in the range 1000–300 cm^−1^. The dried samples were mixed with KBr matrix, and spectra were recorded in transmission mode. TGA of samples were carried out on the dried precipitate by using Perkin Elmer differential thermal analyzer (940 Winter St. Waltham, MA, 02451, USA). Surface area and pore size distribution analyses of samples were performed using BET Micrometrics Gemini VII (4356 Communications Drive, Norcross, GA 30093-2901, USA). The dielectric properties and impedance spectroscopy measurements were carried out in the frequency range of 100 Hz to 5 MHz using a Wayne Kerr LCR meter bridge (WK 6500 B) [Durban Road, Bognor Regis, West Sussex, PO22 9QT, UK].

## 3. Results

**Figure 1:**
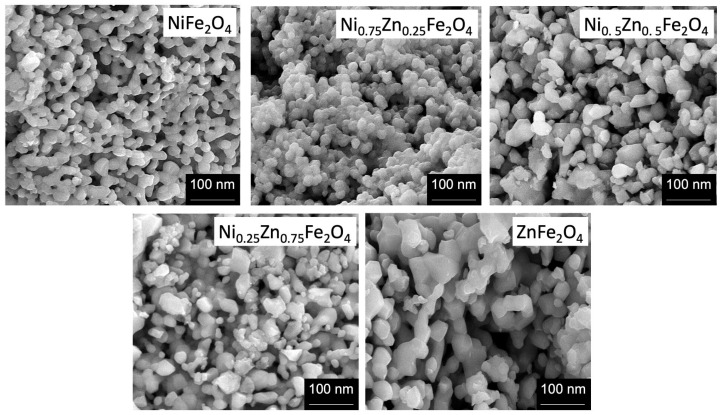
The SEM analysis for Ni_1−x_Zn_x_Fe_2_O_4_ (x = 0, 0.25, 0.5, 0.75, 1) nanoparticles with increasing zinc concentration.

The morphology of Ni_1−x_Zn_x_Fe_2_O_4_ nanoferrite samples was analyzed by using high-resolution scanning electron microscope (SEM), operating at 20 KV. The SEM provides information about the structure of nanoferrites having different compositions. We used powder samples for the morphological analysis. The SEM images depict that nearly all the Ni_1−x_Zn_x_Fe_2_O_4_ nanoparticles exhibit a globular spherical shape and a narrow size distribution, as shown in Figure 1. The particle sharpness is more or less orbicular, possessing few clusters and agglomeration in between the particles. The SEM images indicated that the particle size of the samples lies in the nanometer regime (20–70 nm). The SEM images show that pure nickel ferrite nanoparticles possess spherical symmetry and uniformity. However, with increasing zinc concentration, the morphology of the particles slightly changes. The lower zinc concentration (x = 0.25) did not influence the morphology, but the compactness and agglomeration were slightly enhanced in Figure 1. However, when zinc (x = 0.5) is in equivalent concentration in comparison with nickel, it influences the nanostructure a lot, and an apparent transformation is observable from spherical to non-uniform hexagonal and spherical nanoferrites formation.

At higher zinc concentration, fewer spherical and more corned, irregular, non-symmetric particles and agglomeration are observed as shown in Figure 1. Thus, increase in zinc doping gradually impacts the morphology of nickel ferrites, and noticeable transformation takes place from uniform spherical morphology to irregular non-symmetrical nanostructures. Beside this, oxygen concentration also influence the structural, dielectric, magnetic, and magnetoelectric properties [24].

### 3.1. XRD Analysis

**Figure 2:**
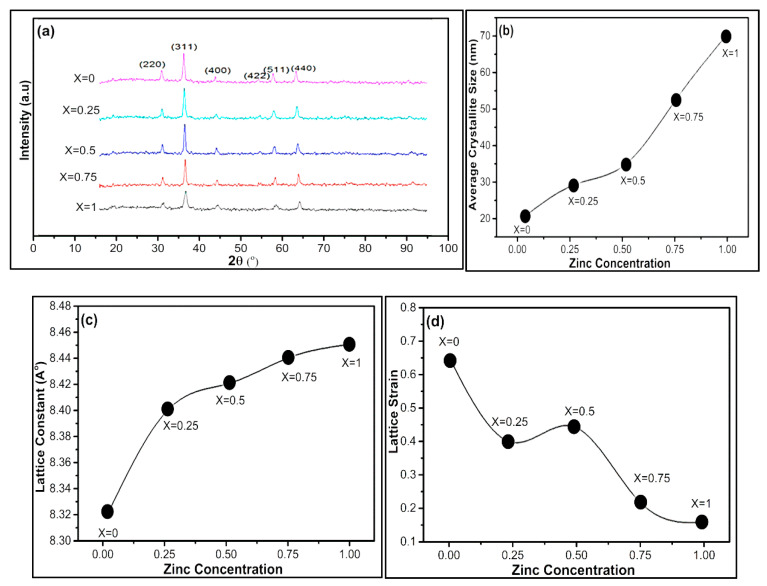
(**a**) The X-Ray diffraction pattern for Ni_1−x_Zn_x_Fe_2_O_4_ nanoferrites (x = 0, 0.25, 0.5, 0.75, 1). The influence of Zn doping on average (**b**) crystallite size, (**c**) lattice constant, and (**d**) lattice strain.

The X-ray diffraction pattern of nickel zinc nanoferrites with composition Ni_1−x_Zn_x_Fe_2_O_4_ (x = 0, 0.25, 0.5, 0.75, 1)) is presented in Figure 2a. The XRD patterns confirm that all the samples exhibit a polycrystalline FCC spinel structure. The crystallite size of Ni_1−x_Zn_x_Fe_2_O_4_ was calculated using the Scherrer formula [47,48,49]. The full width at half maximum of the strongest reflection was used to calculate the crystallite sizes using Scherrer equation as mentioned below.

D = K λ/βcosθ(3)

Here in, D represents the crystallite size, β is the full width at half maximum (FWHM) of the (3 1 1) peak in radian, K is the shape function equivalent to 0.9, λ is the X-ray wavelength, and θ is the diffraction angle. The presence of broad peaks in the spectrum demonstrates that the mean crystallite size of the prepared samples was in the nanometer range. The experimental results demonstrate that the lattice constant “a” increases from 8.32 Å to 8.45 Å with the increase in zinc content in Ni-based ferrites. Previously, Gul et al. [50,51] and Vaidyanathan et al. [37] also described that the lattice constant enhances with Zn substitution. The reason is that Zn^2+^ ions possess larger ionic radius (0.83 Å) in comparison with Ni^2+^ (0.74 Å) and Fe^3+^ (0.65 Å) ions. Consequently, as the concentration of Zn ions upsurges, the lattice constant also rises. The increase in lattice constant obeys the Vegard’s Law. Our experimental result proves that there is a steady increase in the lattice constant with Zn substitution, which specifies that the lattice expands without disturbing the lattice symmetry. The measured density *ρ_m_* and X-ray *ρ_x_* density can be calculated by using the following relation.

*ρ_m_* = m/п r^2^h (4)

In the above equation, *m* represents mass, *r* is the radius, and *h* is the height of a sample.

*ρ_x_* = 8M/NV (5)

N is Avogadro’s number, *M* is the molecular weight of the sample, *V* represents unit cell volume, and 8 is the number of formula units in a cell. Porosity can be determined by using the following relation. The values of lattice constant, lattice strain, theoretical, and apparent density are shown in Table 1.

*ρ* = 1 − *ρ_m_*/*ρ_x_* (6)

The average crystallite size of Ni_1−x_Zn_x_Fe_2_O_4_ nanoferrites (x = 0, 0.25, 0.5, 0.75, 1) was calculated from the X-ray line broadening considering the intense peak corresponding to the (311) plane and using the Scherrer formula. We synthesized all Ni_1−x_Zn_x_Fe_2_O_4_ nanoferrites under similar settings, though the crystallite size for zinc concentration was not the same, perhaps due to preparation circumstances, which might give rise to different ferrite formation rates. The average crystallite size of Ni_1−x_Zn_x_Fe_2_O_4_ nanoferrites (x = 0, 0.25, 0.5, 0.75, 1) lies within the range 21–70 nm as graphically presented in Figure 2b.

Firstly, we calculated the d-values, Miller indices, and then the lattice constant “a.” Lattice parameter for different values of zinc concentration is graphically plotted in Figure 2c. The calculated lattice constant (**a**) identifies the prepared nanoferrites to be cubic spinel. The lattice parameter enhances with an in increase in Zn^+2^ ion substitution. The lattice constant “a” of nickel-based zinc-doped nanoferrites increases from 8.32 Å to 8.45 Å with an increase in zinc content. The Zn^2+^ have larger ionic radius (0.83 Å) as compared to Ni^2+^ (0.74 Å) and Fe^3+^ (0.65 Å) ions. There is a uniform increment in lattice constant with Zn substitution, which demonstrates that the lattice grows without deteriorating the lattice symmetry of the lattice.

It is experimentally observed that with increasing zinc concentration in Ni_1−x_Zn_x_Fe_2_O_4_ nanoferrites, the lattice strain reduces and lattice constant increases. However, in the absence of zinc, there is maximum lattice strain, and an intermediate lattice strain is observed when zinc and nickel are in equal stoichiometric ratio, as shown in Figure 2d. The lattice strain of Ni_1−x_Zn_x_Fe_2_O_4_ nanoferrites was calculated by using William–Hall method (WH) [52,53]. William and Hall suggested a relation between crystallite size (D) and strain (ε) induced broadening given by the following equation.

βcosθ = 0.9λ/D + 4εsinθ(7)

Here, β represents FWHM in radians, λ is X-ray wavelength, 0.9 is shape factor, and ε is induced strain in the crystal [52,54]. Generally, the lattice strain is attained via the slope of the fit through the plot, which is drawn with 4sinθ along the x-axis and βcosθ along the y-axis [53,55].

### 3.2. FT-IR Spectroscopy Analysis

**Figure 3:**
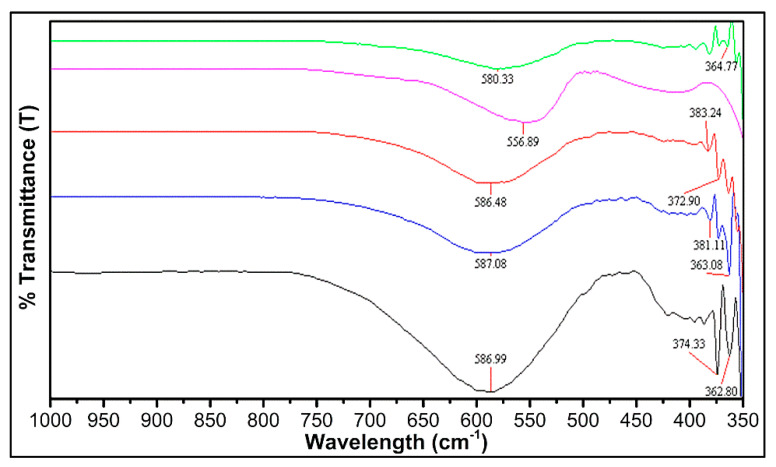
FT-IR spectra of Ni_1−x_Zn_x_Fe_2_O_4_ (x = 0, 0.25, 0.5, 0.75, 1).

IR Spectroscopy is a significant method to analyze the accomplishment of the solid-state reaction and investigate the deformation existence in the spinel structure due to foreign ions or cationic distribution [56,57]. In our experimental results of FT-IR spectra, all the samples exhibit two prominent frequency bands at 370 and 580 cm^−1^, measured in the wavelength range of 1000–350 nm, displaying pure spinel phase. The frequency band at a higher wavelength (ν_1_ (587–557cm^−1^)) is due to the presence of tetrahedral complexes, while the lower frequency bands ν_2_ (383–363cm^−1^) illustrates the octahedral complexes as shown in Figure 3.

The FT-IR analysis shows that the normal vibrational mode of a tetrahedral cluster is greater in comparison with the octahedral cluster. The reason is that the octahedral group possesses longer bond length, while the tetrahedral cluster has a shorter bond length. According to the geometric configuration of ferrites, the metal cations were located nearest to oxygen ions, in two different sublattices of ferrites, i.e., tetrahedral (A-sites) and octahedral sites (B-sites) [58]. The FT-IR spectra of the Ni_1−x_Zn_x_Fe_2_O_4_ ferrites point out two strong absorption bands at 587–557cm^−1^ and 383–363cm^−1^. These bands (ν_1_ and ν_2_) were assigned due to the metal–oxygen ion complexes’ vibrations in the tetrahedral and octahedral positions. The band position differences (ν_1_ and ν_2_) arise due to difference in the Fe^3+^–O^2−^ distance for tetrahedral and octahedral complexes [59]. Generally, the vibrational frequency is dependent on cation mass, bonding force, and cation–oxygen distance [60]. The vibrational frequencies of Ni_1−x_Zn_x_Fe_2_O_4_ ferrites ν_1_ and ν_2_ corresponding to tetrahedral and octahedral metal complexes were mentioned in Table 2.

### 3.3. Thermal Stability

**Figure 4:**
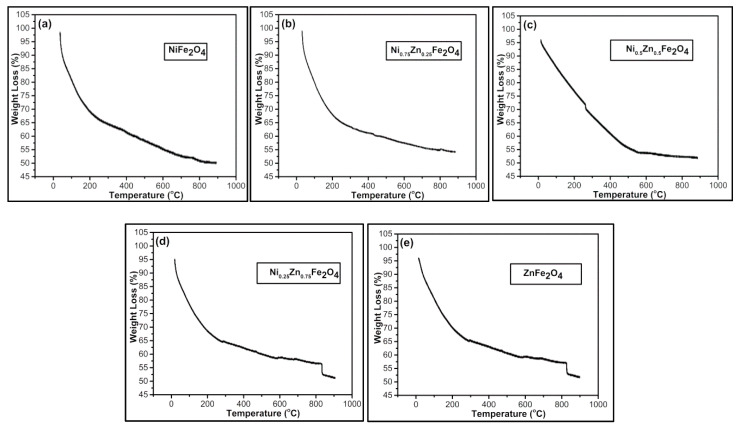
TGA analysis of Ni_1−x_Zn_x_Fe_2_O_4_ (**a**) x = 0, (**b**) x = 0.25, (**c**) x = 0.5, (**d**) x = 0.75, (**e**) x = 1.

The thermal stability of Ni_1−x_Zn_x_Fe_2_O_4_ was studied using a Perkin Elmer differential thermal analyzer. The measurements were performed at room temperature (25 °C) to 1000 °C at a heating rate of 10 °C/min in an oxygen environment. In all samples, the initial weight loss takes place at 120 °C due to vaporization of water molecules from the surface and then at 200 °C from the trapped water. The higher temperature is required to decompose the nitrate network that is obvious from the weight loss at 360 °C. The conversion process took place nearly at 400 °C and converted into ferrite particles at a temperature of 580 °C. At around 850 °C, complete crystallization in cubic spine phase can be seen as shown in Figure 4. Our outcomes were in accordance with the previously reported results [61,62]. Few anomalies were clearly observed at 750 °C, 800 °C, 620 °C, 830 °C, and 820 °C for x = 0, 0.75, 0.5, 0.25, and 1 samples, respectively. The sharp declination observed above 850 °C is due to the residual effect [61]. However, weight loss is not observed over 850 °C showing the formation of only Ni–Zn ferrite nanoparticles in all samples (Figure 4a–e).

### 3.4. BET Surface Area Analysis

**Figure 5:**
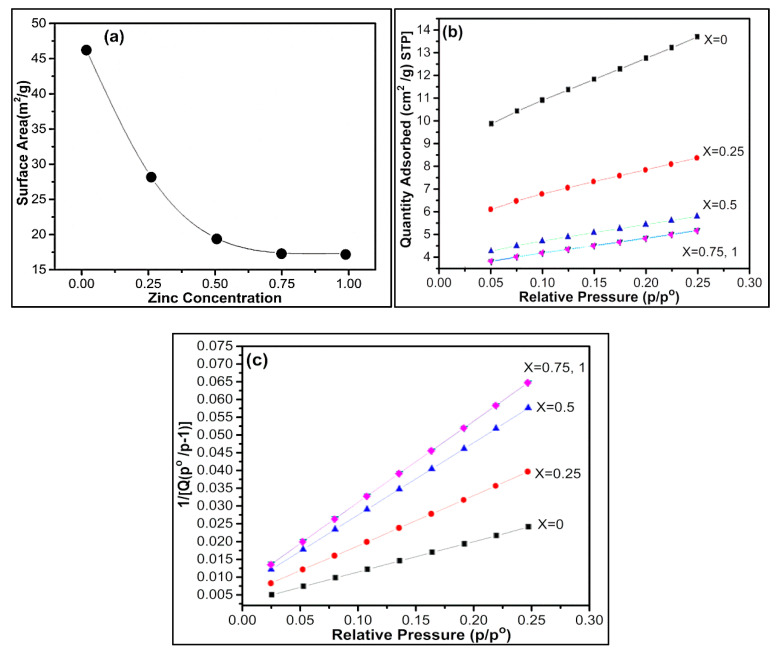
(**a**) A plot of the surface area versus zinc concentration. (**b**) The BET isotherm (linear plot) for Ni_1−x_Zn_x_Fe_2_O_4_. (**c**) BET surface area plot for Ni_1−x_Zn_x_Fe_2_O_4_.

Adsorption of unreactive gas at atomic level plays an important role in determining the surface area, including surface irregularities and pores interior. In BET surface area analysis for nickel zinc ferrites, it is seen that for pure nickel ferrite, we have the maximum surface area, and with the increment of zinc, there is a decrement in surface area as shown in Figure 5a. The surface area reduction may be due to the low adsorption in the presence of zinc. Secondly, the ionic radius of zinc is larger than nickel and lastly arrangement of metal ions. Zinc ions occupy A sites (normal spinel ferrite) while nickel ions occupy B (inverse spinel ferrite) sites, thus affecting the surface area. The specific surface area of nickel zinc ferrites can be theoretically calculated by using the following formula [63,64].

S_BET_ (m^2^/g) = 6000/D_nm_ρ(g/cm^3^) (8)

Here, D_nm_ represents the particle diameter, and ρ represents the density of the material (provided in Supporting Info (Table S1)). It is seen that experimentally calculated surface area (BET) and theoretically calculated surface area values (S-BET) are very close to each other. Equation (9) signifies crystallite size (D_BET_) calculated via BET results through the following formula.

D_BET_ = 6000/S_BET_D_XRD_ (9)

Here in D_XRD_, (*ρ_x_*) represents the calculated density obtained via XRD crystallographic parameters. It is seen that crystallite size determined through XRD results by using Scherer formula in Table 1 and BET results in Table 3 are in agreement with each other.

Figure 5b shows there is maximum adsorption in the case of nickel ferrite, and as nickel decreases, the adsorption rate decreases. There is minimum adsorption for zinc ferrite when x = 0.25 and we get the same curve when x = 0, which shows that adsorption rate is same for pure zinc ferrite and when there is only 0.25 nickel present in nickel zinc ferrite. Figure 5c shows a graph between relative pressure and the inverse of quantity adsorbed, which also proves that there is a minimum adsorption rate for zinc ferrite.

### 3.5. Electrical Properties

Electrical properties include dielectric loss tangent, dielectric constant, or electrical resistivity, which largely depend on size, shape, crystallinity, porosity, and chemical composition of ferrites. Sintering temperature and preparation methods also play an essential role. The hopping process is involved in the conduction in ferrites. The band theory concludes that conductivity is temperature dependent, and change in temperature varies the charge carrier concentration. During the hopping process, the conduction current upsurges via hopping from one iron ion to the next iron ion in B–B sites with temperature increment due to mobility change [65]. However, besides different complex oxides, in perovskites and spinels, the dielectric constant real part (multiferroic substituted hexaferrites) declines gradually at low frequencies and almost monotonically with diamagnetic substitution possessing permeability (real and imaginary parts) peak nearly at 50 GHz determined via the level of diamagnetic substitution [66,67].

#### 3.5.1. Dielectric Constant

The dielectric properties are associated with the electric field distribution within the crystal. The dielectric constant was expressed in real and imaginary parts as mentioned in the following equation:

ε = ε′ − jε″(10)

The real dielectric part gives information about the stored energy. The imaginary part contributes to energy dissipation in the applied AC field.

**Figure 6:**
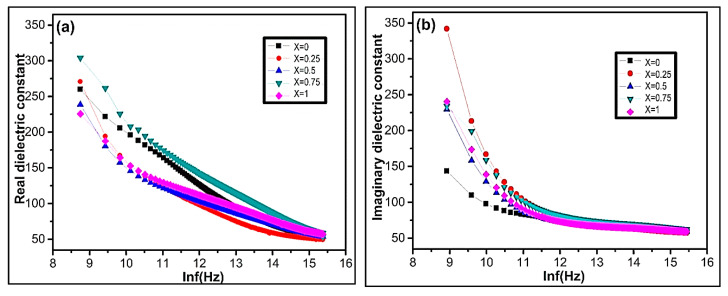
The variation of (**a**) real dielectric constant ε′ and (**b**) imaginary dielectric constant ε″ as a function of lnf(Hz) of Ni_1−x_Zn_x_Fe_2_O_4_.

The dielectric measurements were recorded in the frequency range of 100–5 MHz. Figure 6a,b represents the deviation of real and imaginary parts of dielectric constant with frequency for Ni_1−x_Zn_x_Fe_2_O_4_ ferrite nanoparticles at room temperature. It is experimentally observed that the real and imaginary parts of permittivity exponentially decrease with increasing frequency (Figure 6a,b). At lower frequency, there is a sharp decrement of permittivity in both the real and imaginary parts. However, it remains constant for all compositions with increasing frequency. The dielectric constant deviance with frequency occurs due to space charge polarization and Maxwell Wagner type interfacial polarization [68,69] agreeing on the Koops phenomenological theory [70,71]. The experimental result demonstrates that the polarization decreases with increasing frequency. However, it becomes persistent, which shows the frequency-independent behavior beyond a certain frequency limit. In nanoferrites, the space charge polarization arises due to the inhomogeneous dielectric structure [72]. The nanoferrites comprise of crystalline conducting grains separated by weak-conducting amorphous grain boundaries [73]. In ferrites, dielectric polarization arises due to the electron exchange between Fe^+2^ and Fe^+3^ ions generating the directional field [74]. The electronic exchange between Fe^+2^ and Fe^+3^ ions cannot follow the alternating field with increasing external applied electric field [72,75]. The dielectric constant declines with increasing frequency, as shown in Figure 6. In nanoferrites, the magnitude of electron exchange is dependent on Fe^+2^/Fe^+3^ ions concentration at B site [75].

The experimental result demonstrates that the imaginary part of the dielectric constant is more pronounced in comparison to the real part. The dispersion of dielectric constant is maximum for the sample x = 0.75 (Figure 6b). On the octahedral sites, the maximum dispersion is due to the existence of available Fe^+2^ ions. The sample x = 0.75 possess a higher concentration of ferrous ions at octahedral sites in comparison to other Ni_1−x_Zn_x_Fe_2_O_4_ samples. The sample X = 0.75 shows maximum polarization, which enhances higher permittivity due to electron transfer between Fe^+2^/Fe^+3^ ions.

#### 3.5.2. Dielectric Loss Tangent

**Figure 7:**
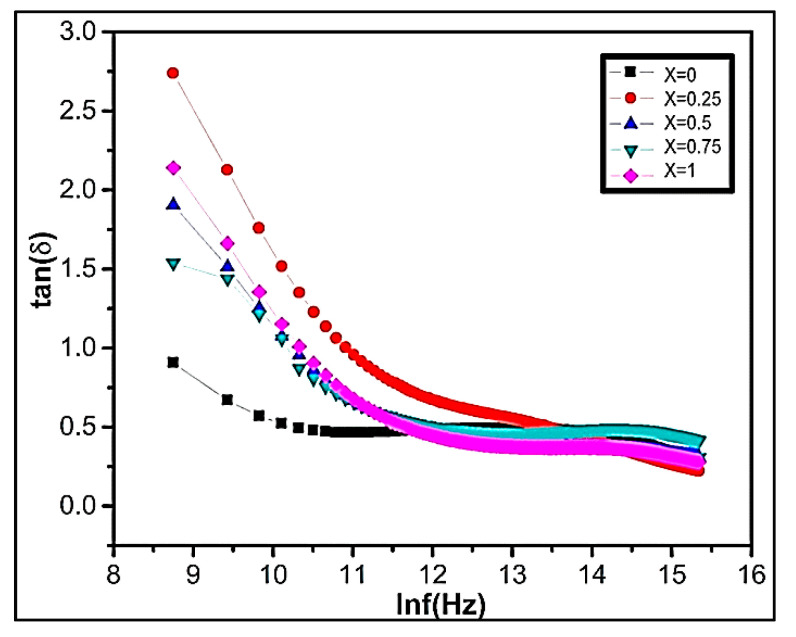
The variation of dielectric loss tangent tan(δ) as a function of lnf(Hz) of Ni_1−x_Zn_x_Fe_2_O_4_.

The dielectric loss tangent explains the energy declines within the ferrite. The polarization lags behind the applied alternating field, when the dielectric loss tangent rises. Figure 7 represents the graph of dielectric loss varying with frequency. The experimental result demonstrates that in all our synthesized samples in the lower frequency region, the dielectric loss factor is high. The dielectric loss is related to grain boundaries. The high dielectric loss is because of higher resistance due to the existence of grain boundaries. It requires efficient energy for electron transfer between Fe^+2^/Fe^+3^ ions, which causes a high loss at a low-frequency region. However, at high-frequency regions, the resistivity declines due to grains. Small energy is needed at octahedral sites for electron transference mechanism between the two iron ions. The high dielectric loss at lower frequencies is due to different factors including impurities, crystal defects, moisture, and inhomogeneity.

#### 3.5.3. AC Conductivity (Ϭ_ac_)

Conductivity explains the conduction mechanism and charge carrier in ferrite materials. Conductivity characterizes an increasing function of frequency when it occurs via electrons springing. In the case of band conduction, frequency shows a decreasing trend. The following equation represents material conductivity:

Ϭ_tot_ = Ϭ_o_(T) + Ϭ(w,T) (11)

**Figure 8:**
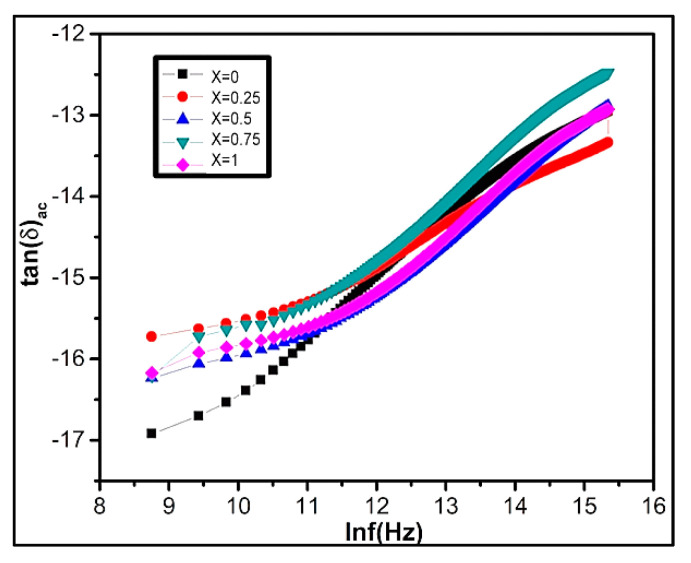
Graphical representation of the variation of tan Ϭ_ac_ conductivity lnf(Hz) of Ni_1−x_Zn_x_Fe_2_O_4_.

Here in, Ϭ_o_(T) signifies DC conductivity that is frequency independent. The expression Ϭ(w,T) characterizes AC conductivity due to electron springing at octahedral situates. The AC conductivity of ferrites can be calculated by using Ϭ_ac_ = ε′ε_o_wtanδ. The variation of lnϬ_ac_ with lnf for Ni_1−x_Zn_x_Fe_2_O_4_ ferrites at room temperature is shown in Figure 8. Conductivity slowly increases at low frequency, while at high-frequency region conduction increases instantaneously due to the hopping of infinite clusters. According to Koop’s theory, at low frequency, the conductivity occurs due to grain boundaries existence while the conductivity at higher frequency takes place due to conducting grains [5,76,77].

#### 3.5.4. Impedance Spectroscopy

**Figure 9:**
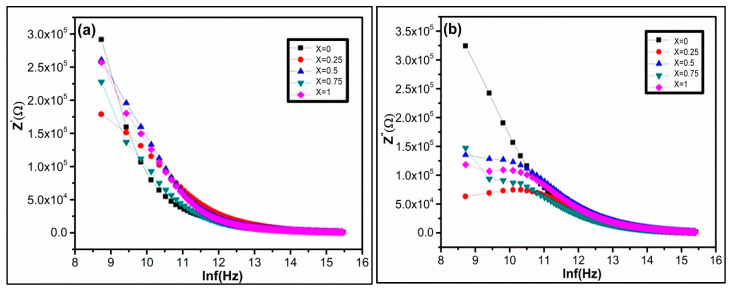
Variation of (**a**) real part and (**b**) imaginary part of impedance z′ as a function of lnf(Hz) of Ni_1−x_Zn_x_Fe_2_O_4._

Impedance spectroscopy associates the material’s dielectric properties with its microstructures. It also helps in analyzing the influence of various factors such as interfaces, grains, or grain boundaries of polycrystalline materials. The impedance measurements (IM) give us statistics regarding resistive and reactive constituents. At room temperature, the IM were performed in the frequency range from 100–5 MHz. Figure 9a demonstrates the graph presenting real part variation of impedance as a function of frequency. The experimental result shows that z’ declines with increasing frequency. As a result, AC conductivity upsurges with applied frequency. Figure 9b shows the variation of the reactive part of impedance as a function of applied frequency at room temperature. It is graphically evident that values of imaginary impedance first increases showing peaking nature, and then start to decrease in the higher frequency region.

#### 3.5.5. Complex Impedance Spectrum Analysis

**Figure 10:**
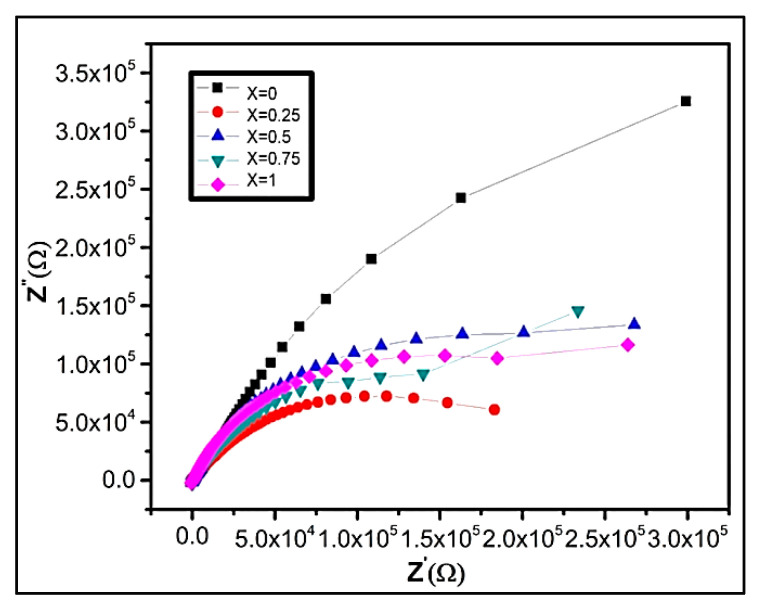
Cole–Cole plot of Ni_1−x_Zn_x_Fe_2_O_4_ samples at room temperature.

Complex impedance gives information regarding the electrical conduction mechanism and the charge transport behavior of nanocrystalline materials. It provides statistics about the impedance, resistive and reactive parts and provides a correlation between the electrical and structural properties of the material [78]. The graphical plot in Figure 10 displays a single semicircle, which is typically associated with the material’s electrical properties. In general, two semicircles may appear, representing different contributions. The first semicircle in the low-frequency region illustrates resistance due to the grain boundary. At the high-frequency region, the second semicircle represents resistance due to grains or bulk properties [73,74,75,76,77,78,79]. At an applied frequency, the complex impedance of grains and grain boundaries can be written as:

z* = z′ + z″(12)

z′ = [{R_g_/(1 + w_g_ C_g_ Rg)^2^} + {R_g_/(1 + w_gb_ C_gb_ R_GB_)^2^}] (13)

z″ = [{R_g_/1 + (w_g_ C_g_ R_g_)^2^} + {R_g_/1 + (w_gb_ C_gb_ R_gb_)^2^}] (14)

Here, R_g_, C_g_, R_gb_, and C_gb_ characterize the resistance and capacitance, while w_g_ and w_gb_ signify semicircles’ peak frequency for grain and grain boundaries, repectively.

The capacitances were estimated from the circular arc maximum height, and the resistances were derived from circular arc intercept on z′ axis. The maximum height of the individual semicircle is evaluated using the following equation:

z′ = −z″(15)

The capacitance at grain and grain boundaries can be calculated by using the following relation:

C_g_ = 1/R_g_w_g_ (16)

C_g_ = 1/R_gb_w_g_ (17)

The relaxation time for grain and grain boundaries can be calculated by:

Ґ_g_ = 1/wg = C_g_R_g_ (18)

Ґ_g_ = 1/w_gb_ = C_gb_R_gb_ (19)

Figure 10 represents cole–cole plot (complex impedance, z′/z″.) for all compositions as a function of frequency. It demonstrates an individual semicircle arc indicating conduction due to grain boundaries. Generally, the impedance measurement displays two overlapped arcs of semicircles contributed by semiconducting grains at high-frequency region and insulating grain boundaries in the low-frequency region. However, at room temperatures, the arcs are not well resolved, but as temperature upsurges the arcs due to grain and grain boundaries become noticeable. In our experimental results, we attain one semicircle, which concludes that maximum conduction is through grain boundary in all Ni_1−x_Zn_x_Fe_2_O_4_ samples [73,80]. Moreover, it may occur also due to transport of charge carriers, conduction band overlapping in bulk, or electrode interfaces [52,81]. The oxygen stoichiometry also significantly affects the electric, magnetic, or magnetoelectric properties of nanoferrites, hexaferrites, and complex oxides [24,82].

## 4. Conclusions

Our experimental results demonstrate that Zn-doped spinel nanoferrites Ni_1−x_Zn_x_Fe_2_O_4_ were efficaciously prepared via the co-precipitated method. The XRD analysis confirms the existence of a single spinel phase in nanoferrites. It was observed that the lattice parameters, density, and grain size of Ni_1−x_Zn_x_Fe_2_O_4_ enhance with the addition of zinc concentration (x). Furthermore, dielectric constant ε and dielectric loss tangent (tan δ) decline with increasing field frequency. The experimental result demonstrates that AC conductivity Ϭ_ac_ also increases with the increasing frequency. The cole–cole plot shows that maximum conduction in nickel zinc ferrite is due to grain boundaries. The surface area analysis verifies that pure nickel ferrite shows maximum surface area. The increment of zinc causes a decrement in the surface area of nanoparticles (Ni_1−x_Zn_x_Fe_2_O_4_).

## Figures and Tables

**Table 1:** Crystallite size, lattice constant, and lattice strain for Ni_1−x_Zn_x_Fe_2_O_4_.

Zinc Concentration	Crystallite Size, D (nm)	Lattice Constant, (Å)	Lattice Strain%	*P_x_* g/cm^3^	*P_x_* g/cm^3^	Porosity %
0	21.3	8.32	0.631	5.40	3.76	31
0.25	30	8.40	0.377	6.71	3.79	44
0.5	35.5	8.42	0.416	6.67	3.81	43
0.75	53.1	8.44	0.215	6.62	3.77	44
1	70.6	8.45	0.162	5.30	3.86	27

**Table 2:** Vibrational frequencies corresponding to tetrahedral and octahedral metal complexes.

X	0	0.25	0.5	0.75	1
Tetrahedral band	586.99	587.08	586.48	556.89	580.30
Octahedral band	374.33	381.11	372.90	383.24	364.77

**Table 3:** The surface area analysis of nickel zinc nanoferrites.

Sr. No	Ni_1−x_Zn_x_Fe_2_O_4_	BET Surface Area m^2^/g	Specific Surface Area S_BET_ (m^2^/g)	D_BET_
1	NiFe_2_O_4_	46.4871	52.1	21.32
2	Ni_0.75_Zn_0.25_ Fe_2_O_4_	28.2980	29.80	30.41
3	Ni_0_._5_Zn_0.5_Fe_2_O_4_	19.5481	25.33	36
4	Ni_0.25_Zn_0.75_Fe_2_O_4_	17.4112	17.06	52.41
5	ZnFe_2_O_4_	17.3383	16.03	66

## Supplementary Materials

The following are available online at https://www.mdpi.com/2079-4991/9/7/1024/s1, Table S1 represents the average particle size variation of nickel zinc nano ferrites estimated via SEM.
