# Correction: Khan et al. Influence of Zn^+2^ Doping on Ni-Based Nanoferrites; (Ni_1−x_ Zn_x_Fe_2_O_4_). *Nanomaterials* 2019, *9*, 1024

**DOI:** 10.3390/nano15120878

**Published:** 2025-06-06

**Authors:** Sadaf Bashir Khan, Syed Irfan, Shern-Long Lee

**Affiliations:** 1 Institute for Advanced Study, Shenzhen University, Shenzhen 518060, China; 2 Key Laboratory of Optoelectronic Devices and Systems of Ministry of Education and Guangdong Province, College of Optoelectronic Engineering, Shenzhen University, Shenzhen 518060, China; 3 Shenzhen Key Laboratory of Advanced Thin Films and Applications, College of Physics and Optoelectronics Engineering, Shenzhen University, Shenzhen 518060, China

## Error in Figure

In the original publication [1], there was a mistake in Figure 1 as published. The authors mistakenly uploaded the identical SEM images for samples with zinc concentrations of 0.5 and 0.75. The corrected Figure 1 appears below.

## Text Correction

There was a typing error in the original publication. The nanoparticle size varies from 20 to 70 nm. The authors mistakenly wrote 60 nm, instead of 70 nm. The correction has been made to the following paragraphs:

A correction has been made to the Abstract:

“Nickel zinc nanoferrites (Ni_1−x_Zn_x_Fe_2_O_4_) were synthesized via a chemical co-precipitation method having stoichiometric proportion (x) altering from 0.00 to 1.00 in steps of 0.25. The synthesized nanoparticles were sintered at 800 °C for 12 h. X-ray diffraction patterns illustrate that the nanocrystalline cubic spinel ferrites have been obtained after sintering. The Scherrer formula is used to evaluate the particle size using the extremely intense peak (311). The experimental results demonstrate that the precipitated particles’ size was in the range of 20–70 nm. Scanning electron microscopy (SEM) is used to investigate the elemental configuration and morphological characterizations of all the prepared samples. FTIR spectroscopy data for respective sites were examined in the range of 300–1000 cm^−1^. The higher frequency band ν_1_ was assigned due to tetrahedral complexes, while the lower frequency band ν_2_ was allocated due to octahedral complexes. Our experimental results demonstrate that the lattice constant a_o_ increases while lattice strain decreases with increasing zinc substitution in nickel zinc nanoferrites”.

A correction has been made to Section 3. Results, Paragraph 1:

“The morphology of Ni_1−x_Zn_x_Fe_2_O_4_ nanoferrite samples was analyzed by using high-resolution scanning electron microscope (SEM), operating at 20 KV. The SEM provides information about the structure of nanoferrites having different compositions. We used powder samples for the morphological analysis. The SEM images depict that nearly all the Ni_1−x_Zn_x_Fe_2_O_4_ nanoparticles exhibit a globular spherical shape and a narrow size distribution, as shown in Figure 1. The particle sharpness is more or less orbicular, possessing few clusters and agglomeration in between the particles. The SEM images indicated that the particle size of the samples lies in the nanometer regime (20–70 nm). The SEM images show that pure nickel ferrite nanoparticles possess spherical symmetry and uniformity. However, with increasing zinc concentration, the morphology of the particles slightly changes. The lower zinc concentration (x = 0.25) did not influence the morphology, but the compactness and agglomeration were slightly enhanced in Figure 1. However, when zinc (x = 0.5) is in equivalent concentration in comparison with nickel, it influences the nanostructure a lot, and an apparent transformation is observable from spherical to non-uniform hexagonal and spherical nanoferrites formation”.

A correction has been made to Section 3. Results, 3.1. XRD Analysis, Paragraph 5:

“The average crystallite size of Ni_1−x_Zn_x_Fe_2_O_4_ nanoferrites (x = 0, 0.25, 0.5, 0.75, 1) was calculated from the X-ray line broadening considering the intense peak corresponding to the (311) plane and using the Scherrer formula. We synthesized all Ni_1−x_Zn_x_Fe_2_O_4_ nanoferrites under similar settings, though the crystallite size for zinc concentration was not the same, perhaps due to preparation circumstances, which might give rise to different ferrite formation rates. The average crystallite size of Ni_1−x_Zn_x_Fe_2_O_4_ nanoferrites (x = 0, 0.25, 0.5, 0.75, 1) lies within the range 21–70 nm as graphically presented in Figure 2b”.

A correction has been made to Section 3. Results, 3.5. Electrical Properties, 3.5.5. Complex Impedance Spectrum Analysis, Paragraph 1:

“Complex impedance gives information regarding the electrical conduction mechanism and the charge transport behavior of nanocrystalline materials. It provides statistics about the impedance, resistive and reactive parts and provides a correlation between the electrical and structural properties of the material [78]. The graphical plot in Figure 10 displays a single semicircle, which is typically associated with the material’s electrical properties. In general, two semicircles may appear, representing different contributions. The first semicircle in the low-frequency region illustrates resistance due to the grain boundary. At the high-frequency region, the second semicircle represents resistance due to grains or bulk properties [73–79]. At an applied frequency, the complex impedance of grains and grain boundaries can be written as:”

The authors state that the scientific conclusions are unaffected. This correction was approved by the Academic Editor. The original publication has also been updated.

## Figures and Tables

**Figure 1:**
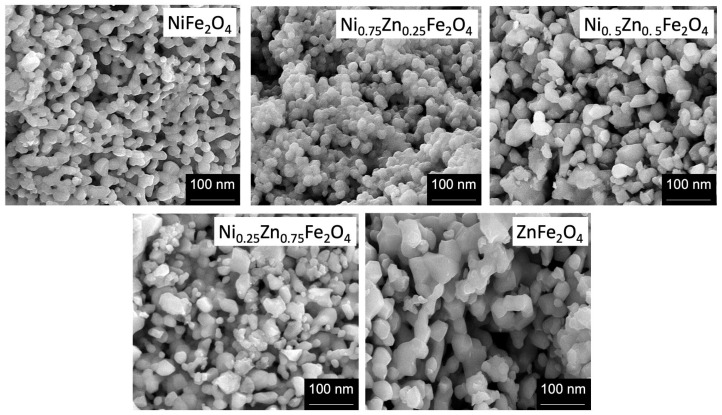
The SEM analysis for Ni_1−x_Zn_x_Fe_2_O_4_ (x = 0, 0.25, 0.5, 0.75, 1) nanoparticles with increasing zinc concentration.
